# The Evoked Potential Score for SSEP and BAEP—A Prognostic Marker for the Long-Term Neurological Outcome in Patients after Poor-Grade Subarachnoid Hemorrhage

**DOI:** 10.3390/diagnostics11061075

**Published:** 2021-06-11

**Authors:** Lisa I. Wadiura, Johannes Herta, Mario Mischkulnig, Dorian Hirschmann, Martin Borkovec, Arthur Hosmann, Andrea Reinprecht

**Affiliations:** 1 Department of Neurosurgery, Medical University of Vienna, 1090 Wien, Austria; johannes.herta@meduniwien.ac.at (J.H.); mario.mischkulnig@meduniwien.ac.at (M.M.); dorian.hirschmann@meduniwien.ac.at (D.H.); arthur.hosmann@meduniwien.ac.at (A.H.); andrea.reinprecht@meduniwien.ac.at (A.R.); 2 Department of Statistics, Ludwig-Maximilians-University Munich, 80539 München, Germany; martin.borkovec@skyforge.at

**Keywords:** subarachnoid hemorrhage, aneurysm, neurorehabilitation, somatosensory evoked potentials, brainstem auditory evoked potentials, evoked potential score

## Abstract

Objective: Evoked potentials are widely used in comatose patients to evaluate neurological function; however, prognostic relevance in patients after SAH is barely investigated. Therefore, we aimed to investigate the prognostic value of the proposed Evoked Potential Score (EPS) for somatosensory (SSEP) and brainstem auditory evoked potentials (BAEP) on the neurological outcome in patients after poor-grade SAH. Methods: We retrospectively analyzed patients after poor grade SAH (Hunt and Hess (HH) grade IV and V) that were admitted to the ICU at the Department of Neurosurgery, MUV, between 2014 and 2017. Measurements of SSEP and BAEP were evaluated separately as well as in a combined model, using the EPS at admission and before ventilator weaning and correlated with the grade of the modified ranking scale at the last available follow up. Results: In total, 48 patients after SAH HH IV/V were included in this study. The EPS for SSEP at admission (*p* = 0.007) and both the EPS for SSEP (*p* = <0.0001) and BAEP (*p* = 0.036) before ventilator weaning were significant prognostic markers for neurological improvement at a mean follow-up period of 14.1 months. In addition, the combined model of the EPS for SSEP/BAEP performed as a prognostic marker for neurological improvement (“at admission” *p* = 0.007; “before ventilator weaning” *p* < 0.001). Conclusions: In the first series to date we found a high prognostic significance for the EPS as a combined model, as well as a separate analysis for SSEP and BAEP in patients after SAH IV and V. In the future, these findings potentially support physicians in ethically challenging decision-making processes and in advice for patients’ families under consideration of an individual evaluation of each patient.

## 1. Introduction

Patients with poor-grade subarachnoid hemorrhage (SAH) have commonly been reported to suffer from poor prognosis. Since the late 1990s favorable outcome has been reported frequently, in approximately 30% of cases. This improvement in outcome might be based on novel techniques of aneurysm repair and improved methods of neurorehabilitation [1,2,3,4]. The initial clinical evaluation of patients with poor-grade SAH is often aggravated due to the need for sedative and analgesic medical treatment or the presence of epileptic seizures, and might lead to underestimation of potential recovery. A few studies focused on the analysis of clinical outcome beyond the first 6 months after hemorrhage [4,5]. In a review of the Barrow ruptured aneurysm trial analyzing the long-term outcome of 88 poor-grade SAH patients the authors reported, that 19% of their cohort improved at least one grade in the modified ranking scale (mRS) between one and three years after bleeding [5]. Based on these results, it can be assumed that a number of patients might experience neurological improvement over time after severe SAH. The evaluation of neurological function via the measurement of somatosensory evoked potentials (SSEP) and brainstem auditory evoked potential (BAEP) is widely used in various fields of application [6,7,8]. This neurophysiological technique is available in most neuro-intensive care units and can be performed repeatedly as a bedside assessment without additional risk to the patient. The measurements of SSEP and BAEP and their predictability of outcome have been reported in multiple studies concerning unconscious or comatose patients due to cardiac arrest or stroke [6,7,9]. In a recent study analyzing the value of SSEP in patients suffering from SAH, the authors reported that SSEP measurements two weeks after SAH (HH grade III-V) were able to predict clinical improvement up to 6 months of follow up [10]. So far, the prognostic relevance of SSEP in combination with BAEP after poor-grade SAH HH IV and V has not been investigated.

The aim of this study was to retrospectively analyze the prognostic significance of SSEP in combination with BAEP on functional neurological outcome in patients after poor grade SAH. For this purpose, we designed the Evoked Potential Score (EPS) as a modification of the SSEP grading system by Houlden et al. [11]. As a next step, we performed a correlation analysis of the EPS for SSEP and BAEP separately and in a combined model at two different time points, with long-term clinical outcome based on the modified ranking scale (mRS) in patients after poor-grade SAH (HH grade IV and V).

## 2. Materials and Methods

### 2.1. Collection of Clinical Data

In this study, we screened our databank for patients suffering from poor-grade aneurysmal SAH (HH grade IV and V), admitted to the neurosurgical Intensive Care Unit (ICU) at the Department of Neurosurgery, Medical University of Vienna (MUV) from 2014 to 2017. We focused exclusively on poor-grade SAH patients since this patient group is generally deeply sedated at time of admission to the ICU and neurophysiological assessment of SSEP and BAEP requires adequate anesthesia to provide diagnostic results [12]. Patients were included in this study only if aneurysm bleeding occurred ≤3 days prior to admission to the ICU. In order to include a homogenous group of patients we excluded patients with non-aneurysmal SAH due to the fact that these patients differ to a great extent in the origin of the intracranial bleeding from patients with an aneurysmal SAH. Diagnosis of aneurysmal SAH was confirmed by CT and CTA in all patients and additionally by angiography in 29 patients (60%). Patient’s characteristics including sex, age, HH grade at admission, presence of intracerebral hemorrhage (ICH), volume of ICH (volume in mL, measured by Brainlab©, München, Germany), aneurysm location, cause of death, treatment modality (microsurgical clipping, endovascular coiling, conservative treatment), and the need for decompressive hemicraniectomy, external ventricular drainage or shunt placement, as well as tracheotomy, were also analyzed.

According to our strategy and under consideration of an adequate mean arterial blood pressure, all patients received oral nimodipine for 21 days after the hemorrhage. Screening for vasospasm was performed by transcranial Doppler ultrasonography on a daily basis. If mean flow velocity increased significantly (>180 ms), a computed tomography angiography (CTA) and/or a conventional cerebral angiography was performed. The presence of vasospasm was documented based on transcranial Doppler sonography, CTA or angiography. Patients with insufficient documentation were excluded from the study. This retrospective study was approved by the local ethics committee of the Medical University of Vienna (MUV) (EC no.: ECS 1522/2017, amendment).

### 2.2. Acquisition of SSEP and BAEP

According to our strategy, SSEP and BAEP were obtained daily from sedated patients using a NIM-ECLIPSE^®^ monitoring system ver. 4.5.354 (Medtronic XOMED Inc., Memphis, TN, USA). In order to register median nerve, SSEP bilateral median nerves were stimulated alternately at each wrist by skin adhesive silver electrodes. A single pulse technique with a duration of 300 µs and a stimulation rate of 4–5 Hz was used. SSEP waves were recorded form subdermal needle electrodes. Recording electrode pairs were placed on the scalp overlying the contralateral somatosensory cortex (C3′ or C4′) and at the Inion (C1R). SSEP recordings were made by averaging results of 200 stimulus presentations at a bandpass of 20–500 Hz. BAEP were recorded using subdermal needle electrodes applied to the earlobes (A1, A2) and referred to Cz with the ground electrodes at Fpz. Both sides (A1-Cz, A2-Cz) were recorded simultaneously. Clicks of 300 µs and up to 135 dB intensity were used through in-ear plugs at a rate of 36 Hz. A mask of 60 dB intensity was used in the contralateral ear. The bandpass filter was set between 50–1500 Hz and analysis time was 15 ms. At least two runs of 1000 stimuli were averaged for both right and left ear stimulations. Reproducibility was assessed by superimposing the traces on the screen. In our daily routine, neurophysiological technicians perform electrophysiological measurements, including evoked potentials, at our neurosurgical ICU. Medical reports of the neurophysiological measurements are written and confirmed by neurosurgeons specialized in neurophysiology. In our daily practice at the ICU, we routinely rule out local disturbances before measuring and interpreting SSEP and BAEP. We further routinely discuss the presence of comorbidities that eventually effect SSEP and BAEP measurements with the patients’ families whenever possible.

### 2.3. Analysis of SSEP and BAEP

SSEP responses were considered as absent when the cervical control was normal but the N20 and P25 waveform were missing. BAEP responses were considered as absent when the waveform I, III and V were missing. SSEP responses were considered as abnormal when (a) N20 amplitude was less than 0.9 µV or at least 40% lower than the contralateral N20; (b) the N13–N20 interpeak latency was longer than 7.2 ms. BAEP responses were considered as abnormal when (a) peak V amplitude was 50% lower than the contralateral peak; (b) the I–III interpeak latency was longer than 2.5 ms and/or the I–V interpeak latency was longer than 4.5 ms; (c) at least one of the waveforms III or V was not present [13].

In our department SSEP and BAEP measurements are routinely performed on a daily basis in patients suffering from poor-grade SAH. Measurements start from the day of admission to the ICU and stop before the beginning of ventilator weaning due to potential inaccuracy in performance in waking patients. Therefore, the first timepoint of SSEP and BAEP measurements was defined as “at admission”, describing the first neurophysiological examination after admission to the ICU (≤24 h). The second timepoint was defined as “before ventilator weaning”, describing the last SSEP and BAEP measurement performed before weaning from sedation.

### 2.4. EPS—A Modified Version of the SSEP Grading System by Houlden et al., 2010

In order to include both SSEP and BAEP into a prognostic score for the neurological outcome on activity level in patients after poor grade SAH, we designed the EPS based on the SSEP grading system by Houlden et al. [11].

**Figure 1:**
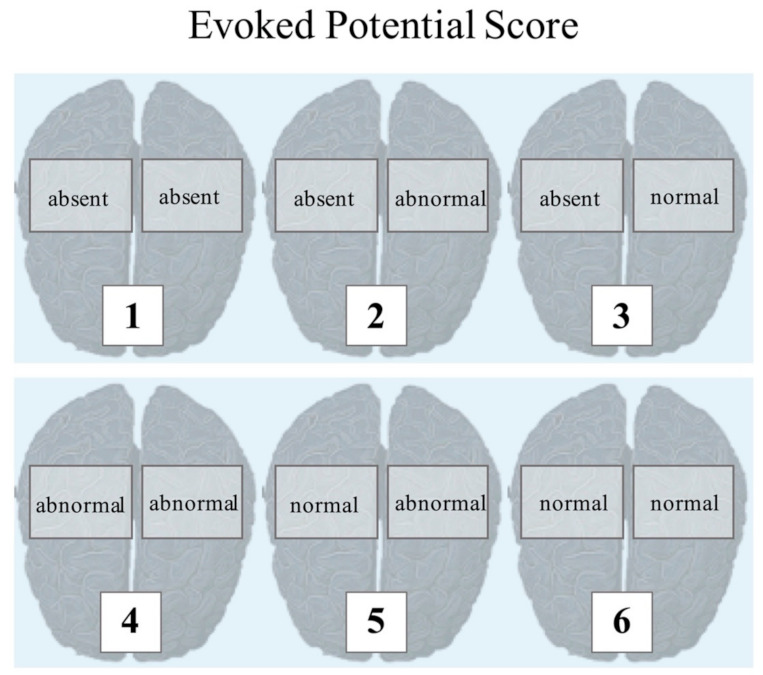
SSEP and BAEP responses from both sites were evaluated separately. In detail, depending on the rating based on the recorded response, an EPS score in the range of 0–6 points was assigned.

SSEP and BAEP responses from both sites were separately evaluated (Figure 1). In detail, depending on the rating based on the recorded response, an EPS score in the range of 0–6 points was assigned.

### 2.5. Separated and Combined Model of EPS for SSEP/BAEP

As a first step, the prognostic significance of neurophysiological monitoring on the neurological outcome regarding level of activities in patients after poor-grade SAH was evaluated. Therefore, we correlated separately the EPS for SSEP and BAEP at two different timepoints (“at admission”, “before ventilator weaning”) with the last available mRS grade during follow up.

In a second step, a combined model of the EPS for SSEP/BAEP was established. In this sense, in each patient the EPS score for SSEP and BAEP was added up separately for both timepoints (“at admission”, “before ventilator weaning”) and the sum was correlated with the last available mRS during follow up.

### 2.6. Evaluation of Clinical Outcome and Neurological Improvement

The diagnostic instrument for the outcome measure for activities was the mRS, which was based on a detailed neurological assessment performed during subsequent hospitalization, neurorehabilitation and/or at appointments at the outpatient clinic. The last available timepoint of follow up was noted for each patient. Patients with an insufficiently documented neurological status were excluded from the study. The mRS grade at the last timepoint of follow up was analyzed. A favorable functional outcome was considered as mRS 0–2; in contrast, an unfavorable outcome was defined as mRS 3–6. Additionally, an analysis of the neurological improvement over time was evaluated comparing the mRS grade at hospital discharge and the mRS grade at the last point of follow up. Improvement, stability or worsening of one or more mRS grades was noted for each patient.

### 2.7. Statistical Analysis

Statistical analyses were performed using SPSS^®^ version 26.0 software (SPSS Inc., Chicago, IL, USA). General statistical characterization of the patient cohort was performed including age, sex, aneurysm localization, HH grade, treatment, presence of ICH and/or intraventricular hemorrhage, external ventricular drainage, ventricular shunt, tracheotomy and infarction. Inferential statistical testing was performed to investigate difference in likelihood of prognostic significance of EPS for SSEP, BAEP and combined SSEP/BAEP on neurological improvement, based on the last available mRS grade during follow up using Spearman correlation. The threshold of statistical significance was set at <0.05.

## 3. Results

According to our databank 173 patients with SAH diagnosis were admitted to the ICU at the Department of Neurosurgery, MUV, between 2014 and 2017. In 50 (29%) of 173 patients an aneurysmal SAH HH IV or V was diagnosed. In 2 (4%) of 50 patients with SAH HH IV/V, a transfer to an external hospital was performed before ventilator weaning. Thus, these two patients were excluded from the study due to lack of data for complete neurophysiological measurements and clinical follow up. In total, 48 patients with poor-grade aneurysmal SAH (29 (60%) patients with HH grade IV, 19 (40%) patients with HH grade V) upon admission at the ICU at the Department of Neurosurgery, Medical University of Vienna between 2014 and 2017 were included in this study.

### 3.1. Patient Characteristics

The median age of this study cohort was 51 years (range 20–77) with a male to female ratio of 1:2. The vast majority of aneurysms were located at the anterior communicating artery (*n* = 17; 35%). In addition to the SAH, 19 (40%) of 48 patients presented with a component of ICH (mean volume: 27.7 mL). Intraventricular hemorrhage occurred in 23 (48%) of 48 cases.

### 3.2. Aneurysm Treatment and Vasospasm

In total 25 of 48 patients were treated by microsurgical aneurysm clipping and 22 of 48 patients underwent endovascular coil embolization. One patient was treated conservatively, after rupture of a basilar aneurysm, due to multiple infarction of the brainstem, missing evoked potentials and herniation signs on the CT one day after admission to ICU. Vasospasm was detected at least once in 30 of 48 patients between day 4 and 21 at the ICU. Further details according to patients’ characteristics are provided in Table 1.

### 3.3. Clinical Outcome and Neurological Improvement during Follow up

Seven of 48 patients died during hospital stay (2 patients with HH IV, 5 patients with HH V). In detail, five patients died during their stay at the ICU due to acute cardiovascular failure following severe brain edema and raised intracranial pressure. In addition, two patients died following cardiorespiratory failure after discharge from the ICU during subsequent hospital stay. Furthermore, nine of 48 patients were lost to follow up after discharge. The mean value of the last available follow up time point in this study cohort was 14.1 months (9.9 +/− 12.1 months). One patient died within the first year. In summary, 20 (42%) of 48 patients presented with a favorable outcome (mRS 0–2) and 28 (58%) of 48 patients with an unfavorable outcome (mRS 3–6) at the last available timepoint of follow up.

In general, patients with mRS < 5 at discharge showed a significantly higher rate of neurological improvement at the last available evaluation timepoint than patients with mRS = 5 (*p* = 0.004). In the subgroup analysis of neurological outcome (mRS), no statistically significant difference (*p* = 0.48) was present between HH grade IV and V patients.

**Figure 2:**
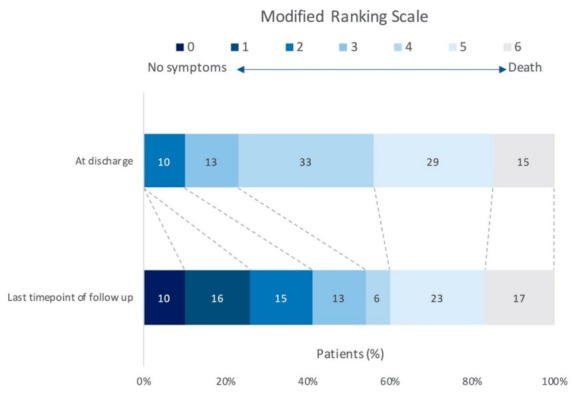
Provides an overview of the neurological improvement in patients (%) after SAH grade IV and V over time. It is of interest that at discharge no patient presented with a mRS of 0 or 1. In contrast, at the last timepoint of follow up 10% of patients had a mRS of 0 and in 16% of cases a mRS of 1 was obtained, respectively.

Detailed analysis of the 41 patients alive after discharge, concerning the mRS grade at discharge compared to the last available mRS grade, showed the following results: 24 (59%) of 41 patients presented with an improvement of at least one mRS grade, seven (17%) of 41 patients were documented as neurologically stable and one patient died during follow up. A detailed documentation of the neurological improvement referred to mRS was noted as follows: five (12%) of 41 patients improved by one mRS grade, 12 (29%) of 41 patients showed an improvement of two mRS grades, six (15%) of 41 patients improved by three mRS grades and one (2%) of 41 patients showed an improvement of four mRS grades. Detailed information on neurological improvement based on mRS grade is provided in Figure 2.

### 3.4. Prognostic Significance of EPS on the Neurological Outcome

- EPS of SSEP measurements

In the whole study group of 48 patients, the EPS for SSEP at admission (ρ = −0.439; *p* = 0.007) as well as the EPS for SSEP before ventilator weaning (ρ = −0.773; *p* < 0.0001) performed as a statistically significant prognostic marker for neurological improvement in patients after poor-grade SAH.

- EPS of BAEP measurements

The analysis of EPS for BAEP at admission correlated well with the last timepoint of follow up, but did not show prognostic relevance (ρ = −0.188; *p* = 0.265). On the contrary, the EPS for BAEP was a statistically significant prognostic marker for neurological improvement before weaning (ρ= −0.371; *p* = 0.036).

### 3.5. Combined Model of the EPS for SSEP/BAEP

**Figure 3:**
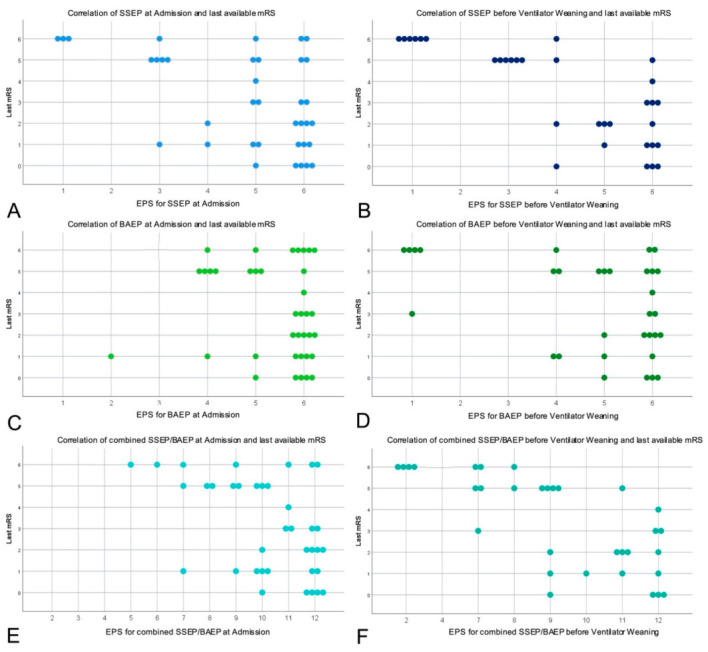
Provides visualization of last available mRS distribution in dependence of separated and combined SSEP and BAEP course, respectively. In detail, correlations of last available mRS and SSEP/BAEP at admission (**A**,**C**) and before respiratory weaning (**B**,**D**) are demonstrated by Scatter plots. Additionally, the combined model of SSEP/BAEP correlated with the last available mRS is demonstrated (**E**,**F**).

In our study, the correlation of the combined model of EPS for SSEP/BAEP with the last available mRS score performed as a statistically significant prognostic marker “at admission” (ρ = −0.433; *p* = 0.007), as well as “before ventilator weaning” (ρ = −0.749; *p* < 0.001) for neurological improvement in patients after poor grade SAH. In order to provide transparency of the data, we include the EPS values and mRS gradings of the whole study group in Table 2. Furthermore, Figure 3 provides visualization of last available mRS distribution in dependence of separated and combined SSEP and BAEP course, respectively.

## 4. Discussion

### 4.1. Prognostic Scores in SAH and Current Drawbacks

SAH after aneurysm rupture remains a life-threatening disease with an overall mortality rate of approximately 30% [14] and is further responsible for 27% of all stroke-related years of potential life lost before age 65 [15]. Prognostic factors for patients suffering from poor-grade aneurysmal SAH are of major importance to indicate the most suitable treatment strategy and to avoid undertreatment. However, prognostic scores in this patients’ group are discussed controversially in the literature [16]. Grading systems such as Hunt and Hess or the World Federation of Neurosurgical societies [17,18] are based on a baseline neurological assessment at the time of admission. Therefore, these scores can easily be influenced by prior epileptic seizures or sedative medication and might lead to an under-grading of prognosis.

### 4.2. Evoked Potentials in SAH and Prognostic Significance

Monitoring of evoked potentials is widely used in comatose patients admitted to neurocritical care to estimate neurological function; however, the prognostic significance in SAH patients has barely been studied. Recently, in the study of Mende et al., the authors found a predicting value for SSEP measured two weeks after hemorrhage in patients suffering from SAH HH grade III-V [10]. However, so far the prognostic significance of SSEP in combination with BAEP in a group of poor-grade SAH patients has not been investigated. A simple accessible scoring system including information from neurophysiological monitoring would be of major importance to support the estimation of neurological outcome in these patients.

### 4.3. Present Study

On that account, we designed the present study to investigate the prognostic significance of the EPS for SSEP in combination with BAEP on the long-term neurological outcome in patients after poor grade SAH. We aimed to focus exclusively on poor grade SAH patients (HH grade IV and V) since these patients generally are intubated and sedated at time of ICU admission and thus sufficient evaluation of neurological functions is challenging. In this study we selected two significant timepoints of analysis, namely “at admission—before ventilator weaning”. In our experience the day of admission is very important in order to evaluate the severity of the primary brain damage caused by the subarachnoid hemorrhage itself. Furthermore, the decision to start ventilator weaning in our daily routine at the ICU requires, that the patient is respiratory, hemodynamically and neurologically stable (e.g., ICP, vasospasm, brain edema and infarction). Therefore, we found these two timepoints significant for the patients’ stay at the ICU and also very important for physicians and the patients’ families.

### 4.4. Neurological Outcome

In order to increase the sensibility for a potential neurological improvement in patients after poor-grade SAH that also could be supported by this study’s results, we designed a graphical illustration in Figure 2 demonstrating the neurological development from discharge to the last timepoint of follow up. In our study cohort, in the majority of patients (58%) an unfavorable outcome was present at the last available follow up time point and can be reported in line with prior studies [4]. However, approximately two thirds (59%) of patients presented with an improvement of at least one mRS grade during follow up. Further, it is of interest that 46% of our patients improved by >1 mRS grade over time. Since the last time point of follow up was documented in more than 50% of our patients at least 6 months after bleeding, our results are in line with the study of Wilson et al. [5]. The authors reported a cumulative improvement measured by mRS in 37% of their cohort after 6 months [5]. According to our data and the results of prior studies [4,5], a distinct group of patients after poor-grade SAH shows a considerable neurological improvement over time. Moreover, based on the analysis of our cohort that a mRS grade of below 5 at discharge was a predictor of a significant neurological improvement in comparison with a grade that equals 5, this timepoint of evaluation might represent an adequate reflection of long-term outcome.

### 4.5. Prognostic Significance of EPS for SSEP on the Neurological Outcome

Our data regarding the EPS for SSEP measurement before ventilator weaning are in line with the reports by Mende et al. [10], showing its prognostic value at a comparable timepoint. In contrast, our results showed as well prognostic significance for the EPS for SSEP at the time of admission. In our study, we exclusively included patients after HH grade IV and V bleedings, whereas Mende et al. also included patients after SAH HH grade III. In our experience, HH grade III patients initially present with a more favorable neurological status, but are at high risk for secondary brain damage based on vasospasm and infarction, which is cause of major concern in this group and may lead to a prompt severe neurological aggravation in the early posthemorrhagic period, whereas, in the majority of patients with a SAH HH grade IV and V, the poor clinical condition at time of admission might be affected by the brain injury induced by the bleeding itself [19,20]. From this perspective, clinical homogeneity is represented more precisely in a group exclusively including patients after SAH HH IV and V. Therefore, a lower HH grade at time of admission might influence the diagnostic value of SSEP and might partly explain the difference between the results of Mende et al. [10]. and the present study.

### 4.6. Prognostic Significance of EPS for BAEP on Neurological Outcome

In this study, we analyzed for the first time the prognostic significance of EPS for BEAP in patients with poor-grade SAH on long-term neurological outcome. Our data showed no prognostic relevance for the EPS for BAEP at admission to the ICU; however, EPS for BAEP performed as a prognostic marker for neurological improvement before ventilator weaning. It is of note that the interpretation of BAEP in patients after SAH caused by a ruptured aneurysm of the anterior circulation is limited and supratentorial impairments are more precisely represented by SSEP, since in the minority of our patients a ruptured aneurysm of the posterior circulation was present and thus, the prognostic power might be underestimated. Future studies with a large patient group after rupture of an aneurysm of the posterior circulation are needed to confirm the value of BAEP in this patient group.

In our routine clinical practice, we aim to provide the best therapy available for each individual patient. In order to understand and improve the clinical and neurological situation (e.g., signs of beginning vasospasm, raising ICP) of unconscious patients, we recommend minimal invasive diagnostic techniques such as SSEP and BAEP. However, it is of note, that despite unfavorable SSEP/BAEP patterns a potential favorable neurological outcome has to be considered for each individual case [21]. In our opinion SSEP and BAEP alone do not provide sufficient diagnostic information to change or adapt courses of treatment at our ICU; however, SSEP and BAEP are an important minimal invasive diagnostic tool to monitor neurological function in addition to radiological, neurological and internal examinations.

### 4.7. Combined Model of EPS for SSEP/BAEP and Future Perspectives

In order to establish a score for evoked potentials combining SSEP and BAEP measurements, we modified the SSEP grading system published by Houlden et al. [11]. We proposed the EPS as a prognostic marker on neurological improvement based on SSEP and BAEP measurements in patients after poor grade SAH. According to our data the EPS can be performed separately for SSEP and BAEP or as a combined model for both modalities. Based on our results the combined model of EPS for SSEP/BAEP showed a statistically significant correlation with the last available mRS and therefore for functional outcome. In this sense we propose the combined model of the EPS for SSEP/BAEP as a useful marker to provide estimation of neurological outcome in patients after poor grade SAH.

The present study provides preliminary data to form the basis for future large prospective studies with the aim to confirm our present findings and establish EPS as a prognostic score for neurological long-term outcome in patients after poor-grade SAH. Consequently, EPS potentially will provide additional information beside initial neurological status and neuroradiology to support neurosurgical decision-making and optimize medical advice for patients’ families under consideration of an individual evaluation for each patient.

### 4.8. Study Limitations

(1) This represents a retrospective study with the known shortcomings thereof. (2) Clinical evaluation at time of admission in patients suffering from a SAH can be challenging, due to well-known influencing factors, such as prior epileptic seizures that remained unnoticed by first-aiders and/or relatives, and incipient hydrocephalus. Since grading systems such as those of Hunt and Hess [6] and the World Federation of Neurosurgical Societies [12] are based on the initial neurological evaluation at the time of admission, these scores are discussed controversially in the literature [22]. Therefore, this study might be influenced by the same confounders. (3) Additionally, the strength of our study is limited by the 50% of patients that were lost to follow up at the time point of 12–24 months after hemorrhage. (4) Another potential influencing factor on the results of our study are the inter- and intra-observer variability of the mRS, as described previously [23]. However, user variability represents a common issue of many scales and therefore most studies assessing outcome by the mRS are affected by this limitation. (5) Furthermore, the mRS measures outcome at level of activities (disability) and does not include neuropsychological parameters. Consequently, an advancement in scoring and testing of patients’ outcome is probably necessary to define neurological improvement in our patients. (6) Finally, we routinely rule out that local disturbances and peripheral lesions are present in unconscious patients before measuring and interpreting BAEP; however, a minimal residual risk of undetected disturbances still remains.

## 5. Conclusions

In this study, we analyzed for the first time the prognostic significance for EPS of SSEP in combination with BAEP on long-term outcome in patients after poor-grade SAH HH grade IV and V. According to data analysis, a statistically significant prognostic value for neurological improvement was present for the EPS of SSEP at admission and both for the EPS of SSEP and BAEP before ventilator weaning. Additionally, the combined EPS model performed for both timepoints (“at admission”, “before ventilator weaning”) as a statistically significant marker for neurological improvement. Consequently, these findings indicate a highly prognostic relevance for EPS in patients after SAH IV and V and thus might be able to support ethically challenging decision making, medical advice for patients’ families and planning of early neurorehabilitation.

## Figures and Tables

**Table 1:** Patient characteristics.

General Patient Information		*n*	%
Number of patients		48	(100)
Gender	male:female	1:2	
Age	median (range)	51 years (20–77)	
Hunt and Hess grade			
	IV	29	(60)
	V	19	(40)
Aneurysm localization			
	Anterior communicating artery	17	(35.4)
	Middle cerebral artery	12	(25.0)
	Internal carotid artery	5	(10.4)
	Posterior communicating artery	5	(10.4)
	Anterior cerebral artery	2	(4.2)
	Basilar artery	2	(4.2)
	Posterior cerebral artery	1	(2.1)
	Vertebral artery	1	(2.1)
	Pericallosal artery	1	(2.1)
	Superior cerebellar artery	1	(2.1)
	Anterior interior cerebellar artery	1	(2.1)
Treatment			
	Clip	25	(52)
	Coil	22	(46)
	Conservative treatment	1	(2)
Intracerebral hemorrhage			
	yes	19	(40)
	no	29	(56)
Intraventricular hemorrhage			
	yes	23	(48)
	no	25	(52)
Decompressive hemicraniectomy			
	yes	4	(8)
	no	44	(92)
External ventricular drainage			
	yes	46	(96)
	no	2	(4)
Shunt			
	yes	18	(37)
	no	30	(63)
Tracheotomy			
	yes	17	(35)
	no	28	(58)
	no data	3	(6)
Vasospasm			
	yes	30	(63)
	no	14	(29)
	no data	4	(8)
Infarction day 4–21			
	yes	16	(33)
	no	28	(58)
	no data	4	(9)
Mortality during hospital stay		7	(10)

**Table 2:** Detailed overview on the SSEP, BAEP and mRS values of the study cohort.

Patient ID	Sex	Age	WFNS Score Grade	EPS—SSEP Admission	EPS—SSEP before Ventilator Weaning	EPS—BAEP Admission	EPS—BAEP before Ventilator Weaning	Last Available mRS
1	F	59	5	6	5	6	6	2
2	M	52	4	5	6	6	6	3
3	F	43	4	6	NA	6	NA	3
4	F	54	4	5	3	4	4	5
5	F	77	4	5	5	2	4	1
6	F	68	5	6	6	4	5	5
7	F	52	4	5	6	6	6	3
8	F	72	5	3	3	4	4	5
9	M	26	4	4	4	6	5	2
10	F	52	4	4	NA	6	NA	1
11	F	64	5	5	NA	5	NA	0
12	F	62	5	3	4	5	5	5
13	F	61	4	3	3	6	5	5
14	F	70	4	NA	NA	NA	NA	5
15	M	60	4	6	6	6	5	1
16	F	44	4	6	6	6	6	0
17	F	45	5	NA	NA	NA	NA	4
18	M	54	4	5	6	5	4	1
19	F	48	5	6	6	6	6	2
20	F	44	5	3	NA	6	NA	1
21	M	56	5	1	1	6	6	6
22	F	57	4	3	1	6	6	6
23	F	57	5	NA	NA	NA	NA	3
24	M	48	4	6	6	6	1	3
25	M	60	5	6	6	6	6	0
26	M	39	5	6	4	6	5	0
27	M	50	4	NA	NA	NA	NA	4
28	F	36	4	6	6	6	6	1
29	M	61	4	6	NA	4	NA	1
30	F	69	4	5	6	6	6	4
31	F	54	4	6	5	6	6	2
32	M	51	4	NA	NA	NA	NA	2
33	F	62	5	NA	NA	NA	NA	3
34	F	61	4	6	3	4	6	5
35	F	65	4	3	3	5	6	5
36	F	45	4	6	1	6	1	6
37	M	45	5	1	1	5	1	6
38	F	67	5	1	1	4	1	6
39	M	64	4	NA	NA	NA	NA	5
40	F	82	4	NA	NA	NA	NA	4
41	F	61	4	NA	NA	NA	NA	2
42	F	83	4	NA	NA	NA	NA	5
43	F	69	5	5	3	5	6	5
44	M	47	4	6	6	6	6	0
45	F	56	4	NA	NA	NA	NA	1
46	M	57	5	6	5	6	6	2
47	M	36	5	5	4	6	4	6
48	F	51	5	6	1	6	1	6

WFNS, World Federation of Neurosurgical Societies; EPS, Evoked Potential Score; SSEP, Somatosensory evoked potentials; mRS, modified ranking scale; NA, no data available; BAEP, Brainstem auditory evoked potentials.
